# Bis{2-[(Tri­phenyl­meth­yl)amino]­phen­yl} diselenide aceto­nitrile monosolvate

**DOI:** 10.1107/S1600536814007806

**Published:** 2014-04-12

**Authors:** Adam Neuba, Tobias Schneider, Ulrich Flörke, Gerald Henkel

**Affiliations:** a Universität Paderborn, Warburger Strasse 100, 33098 Paderborn, Germany

## Abstract

The mol­ecular structure of the title compound, C_50_H_40_N_2_Se_2_·C_2_H_3_N, shows a *syn* conformation of the benzene rings bound to the Se atoms, with an Se—Se bond length of 2.3529 (6) Å and a C—Se—Se—C torsion angle of 93.53 (14)°. The two Se-bonded aromatic ring planes make a dihedral angle of 18.42 (16)°. Intra­molecular N—H⋯Se hydrogen bonds are noted. Inter­molecular C—H⋯Se inter­actions give rise to supra­molecular chains extended along [100]. One severely disordered aceto­nitrile solvent mol­ecule per asymmetric unit was treated with *SQUEEZE* in *PLATON* [Spek (2009 ▶). *Acta Cryst.* D**65**, 148–155]; the crystal data take the presence of this mol­ecule into account.

## Related literature   

Due to the importance of seleno­proteins (*e.g*. thio­redoxin reductases and gluta­thione peroxidases) for essential metabolic processes, we have studied organo diselenide systems with N-donor functions with the aim of synthesizing redox-active selenium copper complexes. For the structure of the sulfido compound, see: Tommasi *et al.* (1999 ▶). For related structures of other bis­aryl diselenides, see: Jones & Ramírez de Arellano (1996 ▶); Meyers *et al.* (1995 ▶); Warin *et al.* (1993 ▶); Wojtowicz *et al.* (2003 ▶). 
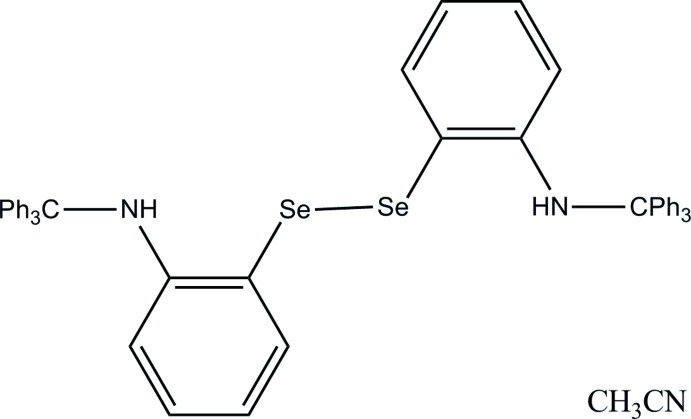


## Experimental   

### 

#### Crystal data  

C_50_H_40_N_2_Se_2_·C_2_H_3_N
*M* *_r_* = 867.81
Triclinic, 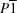

*a* = 9.2364 (16) Å
*b* = 13.245 (2) Å
*c* = 18.248 (3) Å
α = 104.956 (4)°
β = 103.578 (4)°
γ = 101.636 (5)°
*V* = 2013.0 (6) Å^3^
*Z* = 2
Mo *K*α radiation
μ = 1.88 mm^−1^
*T* = 130 K
0.20 × 0.17 × 0.06 mm

#### Data collection  

Bruker SMART APEX diffractometer
Absorption correction: multi-scan (*SADABS*; Sheldrick, 2004 ▶) *T*_min_ = 0.705, *T*_max_ = 0.896
19357 measured reflections
9548 independent reflections
5060 reflections with *I* > 2σ(*I*)
*R*_int_ = 0.059

#### Refinement  

*R*[*F* ^2^ > 2σ(*F* ^2^)] = 0.043
*wR*(*F* ^2^) = 0.068
*S* = 0.64
9548 reflections
495 parameters
2 restraints
H atoms treated by a mixture of independent and constrained refinement
Δρ_max_ = 0.53 e Å^−3^
Δρ_min_ = −0.49 e Å^−3^

### 

Data collection: *SMART* (Bruker, 2002 ▶); cell refinement: *SAINT* (Bruker, 2002 ▶); data reduction: *SAINT*; program(s) used to solve structure: *SHELXTL* (Sheldrick, 2008 ▶); program(s) used to refine structure: *SHELXTL*; molecular graphics: *SHELXTL*; software used to prepare material for publication: *SHELXTL* and local programs.

## Supplementary Material

Crystal structure: contains datablock(s) I, global. DOI: 10.1107/S1600536814007806/tk5302sup1.cif

Structure factors: contains datablock(s) I. DOI: 10.1107/S1600536814007806/tk5302Isup2.hkl

Supporting information file. DOI: 10.1107/S1600536814007806/tk5302Isup3.cml

CCDC reference: 996016

Additional supporting information:  crystallographic information; 3D view; checkCIF report

## Figures and Tables

**Table 1:** Hydrogen-bond geometry (Å, °)

*D*—H⋯*A*	*D*—H	H⋯*A*	*D*⋯*A*	*D*—H⋯*A*
N1—H1⋯Se1	0.88 (1)	2.62 (3)	3.106 (3)	115 (19)
N2—H2⋯Se2	0.89 (2)	2.64 (2)	3.140 (2)	117 (2)
C35—H35*A*⋯Se1^i^	0.95	2.92	3.777 (3)	150

Symmetry code: (i) 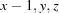.

**Fig. 1.**
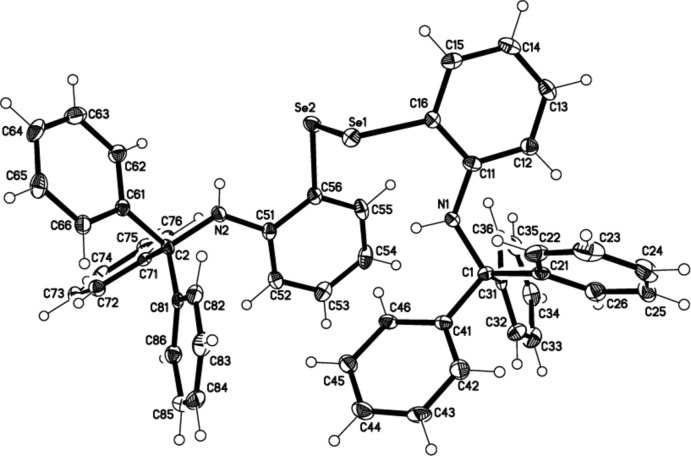
Molecular structure of the title compound. Anisotropic displacement ellipsoids are drawn at the 50% probability level.

## supplementary crystallographic information

### 5. Synthesis and crystallization

The title compound was prepared as follows: 2.08 g (5 mmol) bis­(2-amino­phenyl)­diselenide bis hydro­chloride (Wojtowicz *et al.*, 2003), 2.77 ml tri­ethyl­amine (2.02 g; 20 mmol) and 200 ml of dried aceto­nitrile were placed in a N_2_-flushed 250 ml 2-necked flask. 2.79 g (10 mmol) tri­phenyl­chloro­methane was added and the mixture stirred for one hour. After the solvent was evaporated 100 ml water was added and the mixture was extracted with di­chloro­methane (3 *x* 80 ml). The combined organic layers were dried over Na_2_SO_4_. After filtration the solvent was removed und the crude product was obtained as a yellow powder. For purification, the raw product was stirred in aceto­nitrile (250 ml) for one hour at 80 °C. The hot suspension was filtered. The collected solid was washed with aceto­nitrile and dried under reduced poressure. Yield: 3.5 g (85%). Yellow crystals suitable for X-ray diffraction were obtained by diffusion of Et_2_O into a cold saturated MeCN solution.

Spectroscopic analyses.

^1^H-NMR: (500 MHz, 25 °C, CDCl_3_, δ [p.p.m.]) 6.15 (dd, ^3^J_HH_= 8.3 Hz, ^4^J_HH_= 1.1 Hz, 2H, 6.27) (ddd, ^3^J_HH_= 7.4 Hz, ^3^J_HH_= 7.4 Hz, ^4^J_HH_= 1.1 Hz, 2H); 6.56 (s, 2H,NH); 6.75 (ddd, ^3^J_HH_= 8.3 Hz, ^3^J_HH_= 7.4 Hz, ^4^J_HH_=1.5 Hz 2H); 7.19–7.39 (m, 32 H)

^13^C-NMR: (125 MHz,, 25 °C, CDCl_3_δ [p.p.m.]): 71.7 (C_q_); 115.1 (CH); 116.3 (C_q_); 116.9 (CH); 126.8 (CH); 128.1 (CH); 129.1 (CH); 130.4 (CH); 138.2 (2 C,CH) 145.2 (C_q_)

^15^N-NMR (50,7 MHz,, 25 °C, CDCl_3_δ [p.p.m.]): 101 (N)

IR (KBr, ν, [cm^-1^]): 2850 s, 2604 s, 1945w, 1602*m*, 1564*m*, 1500*m*, 1467 s, 1301w, 1191w, 1122*m*, 1051w, 756 s, 543w, 507w, 440 s

EI—MS (m/*z* (%)): 828.1 (1) [*M*^+^], 243.0 (100) [C(Ph)_3_]^+^; 165.02 (79) [C(Ph)(C_6_H_4_)]^+^; 183.0 (26) [NH_2_C(Ph)_2_]^+^; 105.0 (24)[NH_2_C(Ph)]^+^.

Elemental analysis (*M* = 826.79 g mol^-1^): calcd. for C_50_H_40_N_2_Se_2_: C: 72.64; H: 4.48; N: 3.39; found C: 71.81, H: 4.94, N: 3.47.

### 6. Refinement

Hydrogen atoms were clearly identified in difference syntheses, refined at idealized positions riding on the carbon atoms with isotropic displacement parameters *U*_iso_(H) = 1.2U(C_eq_) and C—H 0.95 Å. H(N) atom positions were refined with N—H = 0.90±0.01 Å, the *U*_iso_ were refined freely. One severely disordered aceto­nitrile solvent molecule per asymmetric unit was treated with the *SQUEEZE* facility in *PLATON* (Spek, 2009) which gave a void count of 43 electrons in the unit cell.

### Crystal data

**Table:**

C_50_H_40_N_2_Se_2_·C_2_H_3_N	*Z* = 2
*M**_r_* = 867.81	*F*(000) = 888
Triclinic, *P*1	*D*_x_ = 1.432 Mg m^−^^3^
Hall symbol: -P 1	Mo *K*α radiation, λ = 0.71073 Å
*a* = 9.2364 (16) Å	Cell parameters from 1733 reflections
*b* = 13.245 (2) Å	θ = 2.3–20.0°
*c* = 18.248 (3) Å	µ = 1.88 mm^−^^1^
α = 104.956 (4)°	*T* = 130 K
β = 103.578 (4)°	Prism, yellow
γ = 101.636 (5)°	0.20 × 0.17 × 0.06 mm
*V* = 2013.0 (6) Å^3^	

### Data collection

**Table:**

Bruker SMART APEX diffractometer	9548 independent reflections
Radiation source: sealed tube	5060 reflections with *I* > 2σ(*I*)
Graphite monochromator	*R*_int_ = 0.059
φ and ω scans	θ_max_ = 27.9°, θ_min_ = 1.7°
Absorption correction: multi-scan (*SADABS*; Sheldrick, 2004)	*h* = −12→12
*T*_min_ = 0.705, *T*_max_ = 0.896	*k* = −17→17
19357 measured reflections	*l* = −24→21

### Refinement

**Table:**

Refinement on *F*^2^	Primary atom site location: structure-invariant direct methods
Least-squares matrix: full	Secondary atom site location: difference Fourier map
*R*[*F*^2^ > 2σ(*F*^2^)] = 0.043	Hydrogen site location: difference Fourier map
*wR*(*F*^2^) = 0.068	H atoms treated by a mixture of independent and constrained refinement
*S* = 0.64	*w* = 1/[σ^2^(*F*_o_^2^) + (0.0025*P*)^2^] where *P* = (*F*_o_^2^ + 2*F*_c_^2^)/3
9548 reflections	(Δ/σ)_max_ = 0.001
495 parameters	Δρ_max_ = 0.53 e Å^−^^3^
2 restraints	Δρ_min_ = −0.49 e Å^−^^3^

### Special details

**Table:**

Geometry. All e.s.d.'s (except the e.s.d. in the dihedral angle between two l.s. planes) are estimated using the full covariance matrix. The cell e.s.d.'s are taken into account individually in the estimation of e.s.d.'s in distances, angles and torsion angles; correlations between e.s.d.'s in cell parameters are only used when they are defined by crystal symmetry. An approximate (isotropic) treatment of cell e.s.d.'s is used for estimating e.s.d.'s involving l.s. planes.
Refinement. Refinement of *F*^2^ against ALL reflections. The weighted *R*-factor *wR* and goodness of fit *S* are based on *F*^2^, conventional *R*-factors *R* are based on *F*, with *F* set to zero for negative *F*^2^. The threshold expression of *F*^2^ > σ(*F*^2^) is used only for calculating *R*-factors(gt) *etc*. and is not relevant to the choice of reflections for refinement. *R*-factors based on *F*^2^ are statistically about twice as large as those based on *F*, and *R*- factors based on ALL data will be even larger.

### Fractional atomic coordinates and isotropic or equivalent isotropic displacement parameters (Å^2^)

**Table:**

	*x*	*y*	*z*	*U*_iso_*/*U*_eq_	
Se1	0.27997 (4)	0.00117 (3)	0.12906 (2)	0.02063 (9)	
Se2	0.16179 (4)	0.11762 (3)	0.07419 (2)	0.02247 (9)	
N1	0.0981 (3)	−0.0249 (2)	0.24933 (15)	0.0194 (6)	
H1	0.182 (2)	0.0250 (18)	0.2542 (17)	0.023 (10)*	
N2	0.3766 (3)	0.3529 (2)	0.17496 (16)	0.0200 (7)	
H2	0.391 (3)	0.2987 (16)	0.1392 (13)	0.016 (9)*	
C1	0.0733 (4)	−0.0128 (3)	0.32780 (18)	0.0178 (8)	
C2	0.5110 (3)	0.4497 (2)	0.21235 (18)	0.0156 (7)	
C11	0.0380 (4)	−0.1129 (3)	0.18084 (18)	0.0181 (8)	
C12	−0.0872 (4)	−0.2010 (2)	0.16825 (19)	0.0206 (8)	
H12A	−0.1357	−0.2014	0.2087	0.025*	
C13	−0.1421 (4)	−0.2869 (3)	0.09890 (19)	0.0239 (8)	
H13A	−0.2287	−0.3451	0.0920	0.029*	
C14	−0.0739 (4)	−0.2906 (3)	0.03873 (19)	0.0244 (8)	
H14A	−0.1117	−0.3511	−0.0088	0.029*	
C15	0.0500 (4)	−0.2048 (3)	0.04903 (18)	0.0202 (8)	
H15A	0.0981	−0.2067	0.0082	0.024*	
C16	0.1061 (3)	−0.1151 (2)	0.11865 (18)	0.0154 (7)	
C21	0.0682 (4)	−0.1204 (3)	0.34699 (18)	0.0197 (8)	
C22	0.1758 (4)	−0.1756 (3)	0.33070 (19)	0.0283 (9)	
H22A	0.2518	−0.1458	0.3091	0.034*	
C23	0.1743 (5)	−0.2727 (3)	0.3454 (2)	0.0399 (11)	
H23A	0.2475	−0.3101	0.3330	0.048*	
C24	0.0652 (5)	−0.3158 (3)	0.3784 (2)	0.0413 (11)	
H24A	0.0635	−0.3827	0.3887	0.050*	
C25	−0.0394 (4)	−0.2612 (3)	0.3960 (2)	0.0339 (10)	
H25A	−0.1127	−0.2899	0.4195	0.041*	
C26	−0.0395 (4)	−0.1646 (3)	0.38004 (19)	0.0261 (9)	
H26A	−0.1140	−0.1282	0.3918	0.031*	
C31	−0.0729 (4)	0.0236 (2)	0.33063 (19)	0.0186 (8)	
C32	−0.0959 (4)	0.0684 (3)	0.40307 (19)	0.0243 (8)	
H32A	−0.0177	0.0793	0.4510	0.029*	
C33	−0.2307 (4)	0.0976 (3)	0.4072 (2)	0.0307 (9)	
H33A	−0.2442	0.1279	0.4575	0.037*	
C34	−0.3450 (4)	0.0826 (3)	0.3381 (2)	0.0291 (9)	
H34A	−0.4392	0.0999	0.3404	0.035*	
C35	−0.3209 (4)	0.0420 (3)	0.2653 (2)	0.0264 (9)	
H35A	−0.3968	0.0345	0.2175	0.032*	
C36	−0.1876 (4)	0.0125 (2)	0.26208 (19)	0.0194 (8)	
H36A	−0.1736	−0.0162	0.2117	0.023*	
C41	0.2175 (4)	0.0787 (3)	0.38798 (18)	0.0181 (8)	
C42	0.3011 (4)	0.0692 (3)	0.4582 (2)	0.0296 (9)	
H42A	0.2694	0.0049	0.4711	0.036*	
C43	0.4303 (4)	0.1517 (3)	0.5100 (2)	0.0328 (10)	
H43A	0.4853	0.1443	0.5584	0.039*	
C44	0.4787 (4)	0.2441 (3)	0.4911 (2)	0.0292 (9)	
H44A	0.5694	0.2995	0.5256	0.035*	
C45	0.3956 (4)	0.2567 (3)	0.42217 (19)	0.0251 (8)	
H45A	0.4281	0.3211	0.4094	0.030*	
C46	0.2639 (4)	0.1745 (3)	0.37143 (18)	0.0206 (8)	
H46A	0.2050	0.1842	0.3248	0.025*	
C51	0.2541 (4)	0.3238 (3)	0.20419 (19)	0.0180 (8)	
C52	0.2299 (4)	0.3938 (3)	0.26885 (18)	0.0216 (8)	
H52A	0.3049	0.4619	0.2981	0.026*	
C53	0.0977 (4)	0.3651 (3)	0.2909 (2)	0.0249 (8)	
H53A	0.0844	0.4136	0.3356	0.030*	
C54	−0.0151 (4)	0.2677 (3)	0.2494 (2)	0.0276 (9)	
H54A	−0.1066	0.2498	0.2641	0.033*	
C55	0.0084 (4)	0.1967 (3)	0.18580 (19)	0.0236 (8)	
H55A	−0.0685	0.1296	0.1565	0.028*	
C56	0.1419 (4)	0.2218 (2)	0.16399 (18)	0.0181 (8)	
C61	0.6142 (4)	0.4383 (2)	0.15633 (19)	0.0175 (8)	
C62	0.5446 (4)	0.4133 (3)	0.07474 (19)	0.0241 (8)	
H62A	0.4357	0.4012	0.0548	0.029*	
C63	0.6328 (4)	0.4056 (3)	0.0219 (2)	0.0311 (9)	
H63A	0.5838	0.3882	−0.0335	0.037*	
C64	0.7904 (4)	0.4235 (3)	0.0502 (2)	0.0300 (9)	
H64A	0.8504	0.4169	0.0144	0.036*	
C65	0.8608 (4)	0.4509 (3)	0.1303 (2)	0.0294 (9)	
H65A	0.9700	0.4645	0.1499	0.035*	
C66	0.7727 (4)	0.4591 (3)	0.1838 (2)	0.0227 (8)	
H66A	0.8230	0.4791	0.2393	0.027*	
C71	0.4658 (4)	0.5555 (3)	0.21059 (18)	0.0169 (8)	
C72	0.5797 (4)	0.6535 (3)	0.24184 (18)	0.0210 (8)	
H72A	0.6831	0.6551	0.2668	0.025*	
C73	0.5466 (4)	0.7494 (3)	0.23759 (19)	0.0244 (8)	
H73A	0.6267	0.8161	0.2598	0.029*	
C74	0.3965 (4)	0.7483 (3)	0.20087 (19)	0.0252 (9)	
H74A	0.3727	0.8141	0.1985	0.030*	
C75	0.2825 (4)	0.6508 (3)	0.16801 (19)	0.0238 (8)	
H75A	0.1797	0.6493	0.1421	0.029*	
C76	0.3160 (4)	0.5551 (3)	0.17223 (18)	0.0186 (8)	
H76A	0.2362	0.4883	0.1488	0.022*	
C81	0.5973 (3)	0.4516 (2)	0.29626 (18)	0.0166 (8)	
C82	0.6575 (4)	0.3647 (3)	0.3028 (2)	0.0229 (8)	
H82A	0.6391	0.3048	0.2566	0.028*	
C83	0.7435 (4)	0.3650 (3)	0.3758 (2)	0.0274 (9)	
H83A	0.7891	0.3074	0.3788	0.033*	
C84	0.7640 (4)	0.4470 (3)	0.4438 (2)	0.0275 (9)	
H84A	0.8235	0.4465	0.4937	0.033*	
C85	0.6982 (4)	0.5298 (3)	0.43931 (19)	0.0263 (9)	
H85A	0.7086	0.5856	0.4865	0.032*	
C86	0.6159 (4)	0.5324 (3)	0.36573 (19)	0.0224 (8)	
H86A	0.5718	0.5909	0.3633	0.027*	

### Atomic displacement parameters (Å^2^)

**Table:**

	*U*^11^	*U*^22^	*U*^33^	*U*^12^	*U*^13^	*U*^23^
Se1	0.0179 (2)	0.0230 (2)	0.0227 (2)	0.00798 (16)	0.00666 (17)	0.00801 (17)
Se2	0.0271 (2)	0.01750 (19)	0.01792 (19)	0.00341 (16)	0.00054 (17)	0.00554 (16)
N1	0.0179 (17)	0.0189 (16)	0.0158 (15)	−0.0024 (14)	0.0043 (14)	0.0029 (13)
N2	0.0171 (16)	0.0178 (16)	0.0214 (17)	0.0014 (13)	0.0078 (14)	0.0012 (13)
C1	0.0187 (19)	0.0202 (19)	0.0126 (18)	0.0030 (15)	0.0041 (15)	0.0045 (15)
C2	0.0085 (17)	0.0156 (18)	0.0198 (18)	0.0003 (14)	0.0041 (15)	0.0034 (15)
C11	0.0181 (19)	0.0187 (18)	0.0150 (18)	0.0074 (15)	0.0002 (15)	0.0038 (15)
C12	0.0168 (19)	0.0218 (19)	0.0199 (19)	0.0033 (15)	0.0025 (16)	0.0056 (16)
C13	0.018 (2)	0.0194 (19)	0.029 (2)	0.0019 (16)	0.0010 (17)	0.0078 (17)
C14	0.027 (2)	0.0184 (19)	0.020 (2)	0.0085 (17)	−0.0029 (17)	0.0005 (16)
C15	0.027 (2)	0.0224 (19)	0.0156 (18)	0.0152 (17)	0.0048 (16)	0.0081 (16)
C16	0.0182 (19)	0.0133 (17)	0.0168 (17)	0.0076 (14)	0.0014 (15)	0.0090 (14)
C21	0.022 (2)	0.0208 (19)	0.0108 (17)	0.0036 (16)	−0.0013 (15)	0.0029 (15)
C22	0.037 (2)	0.031 (2)	0.0154 (19)	0.0116 (19)	0.0040 (18)	0.0063 (17)
C23	0.056 (3)	0.034 (2)	0.025 (2)	0.026 (2)	−0.005 (2)	0.0059 (19)
C24	0.063 (3)	0.016 (2)	0.030 (2)	0.006 (2)	−0.011 (2)	0.0103 (19)
C25	0.032 (2)	0.028 (2)	0.028 (2)	−0.0073 (19)	−0.0076 (19)	0.0127 (19)
C26	0.022 (2)	0.029 (2)	0.023 (2)	0.0058 (17)	−0.0011 (17)	0.0077 (17)
C31	0.0189 (19)	0.0135 (17)	0.024 (2)	0.0012 (15)	0.0067 (16)	0.0096 (15)
C32	0.026 (2)	0.029 (2)	0.021 (2)	0.0112 (17)	0.0072 (17)	0.0109 (17)
C33	0.038 (2)	0.028 (2)	0.035 (2)	0.0159 (19)	0.020 (2)	0.0126 (19)
C34	0.019 (2)	0.025 (2)	0.049 (3)	0.0107 (17)	0.011 (2)	0.0166 (19)
C35	0.020 (2)	0.022 (2)	0.033 (2)	0.0031 (16)	0.0013 (18)	0.0091 (17)
C36	0.0175 (19)	0.0175 (18)	0.0203 (19)	0.0015 (15)	0.0043 (16)	0.0053 (15)
C41	0.0163 (19)	0.0215 (19)	0.0144 (18)	0.0053 (15)	0.0036 (15)	0.0031 (15)
C42	0.029 (2)	0.030 (2)	0.026 (2)	0.0021 (18)	0.0021 (18)	0.0127 (18)
C43	0.034 (2)	0.039 (2)	0.015 (2)	0.005 (2)	−0.0072 (18)	0.0083 (18)
C44	0.023 (2)	0.031 (2)	0.023 (2)	0.0015 (18)	−0.0036 (17)	0.0048 (18)
C45	0.026 (2)	0.020 (2)	0.025 (2)	0.0036 (17)	0.0049 (18)	0.0059 (17)
C46	0.020 (2)	0.023 (2)	0.0146 (18)	0.0048 (16)	−0.0001 (16)	0.0046 (16)
C51	0.0137 (18)	0.0199 (19)	0.0219 (19)	0.0045 (15)	0.0040 (16)	0.0105 (16)
C52	0.020 (2)	0.0176 (19)	0.027 (2)	0.0032 (15)	0.0068 (17)	0.0076 (16)
C53	0.029 (2)	0.024 (2)	0.031 (2)	0.0153 (17)	0.0148 (18)	0.0144 (18)
C54	0.023 (2)	0.028 (2)	0.047 (2)	0.0133 (18)	0.0199 (19)	0.023 (2)
C55	0.0158 (19)	0.0206 (19)	0.033 (2)	0.0026 (16)	0.0014 (17)	0.0144 (17)
C56	0.0190 (19)	0.0149 (18)	0.0218 (19)	0.0057 (15)	0.0036 (16)	0.0100 (15)
C61	0.0177 (19)	0.0153 (18)	0.0210 (19)	0.0048 (15)	0.0080 (16)	0.0060 (15)
C62	0.021 (2)	0.029 (2)	0.023 (2)	0.0079 (17)	0.0073 (17)	0.0086 (17)
C63	0.039 (3)	0.032 (2)	0.023 (2)	0.0105 (19)	0.0122 (19)	0.0055 (18)
C64	0.034 (2)	0.032 (2)	0.036 (2)	0.0136 (19)	0.025 (2)	0.0148 (19)
C65	0.018 (2)	0.036 (2)	0.041 (2)	0.0088 (18)	0.0114 (19)	0.020 (2)
C66	0.018 (2)	0.025 (2)	0.024 (2)	0.0029 (16)	0.0060 (17)	0.0106 (17)
C71	0.0188 (19)	0.0199 (19)	0.0142 (18)	0.0058 (15)	0.0089 (16)	0.0053 (15)
C72	0.0185 (19)	0.022 (2)	0.0204 (19)	0.0053 (16)	0.0033 (16)	0.0067 (16)
C73	0.025 (2)	0.0169 (19)	0.031 (2)	0.0035 (16)	0.0106 (18)	0.0069 (17)
C74	0.033 (2)	0.028 (2)	0.029 (2)	0.0178 (18)	0.0171 (19)	0.0206 (18)
C75	0.0166 (19)	0.036 (2)	0.021 (2)	0.0104 (17)	0.0073 (16)	0.0109 (18)
C76	0.0164 (19)	0.0226 (19)	0.0176 (18)	0.0040 (15)	0.0052 (15)	0.0088 (16)
C81	0.0135 (18)	0.0157 (18)	0.0210 (19)	−0.0002 (14)	0.0092 (16)	0.0059 (15)
C82	0.025 (2)	0.0206 (19)	0.027 (2)	0.0068 (16)	0.0138 (18)	0.0090 (17)
C83	0.027 (2)	0.028 (2)	0.036 (2)	0.0117 (18)	0.0109 (19)	0.0202 (19)
C84	0.031 (2)	0.033 (2)	0.021 (2)	0.0054 (18)	0.0043 (17)	0.0161 (18)
C85	0.029 (2)	0.024 (2)	0.020 (2)	0.0024 (17)	0.0054 (17)	0.0029 (17)
C86	0.024 (2)	0.0188 (19)	0.025 (2)	0.0058 (16)	0.0091 (17)	0.0071 (16)

### Geometric parameters (Å, º)

**Table:**

Se1—C16	1.922 (3)	C43—C44	1.372 (4)
Se1—Se2	2.3529 (6)	C43—H43A	0.9500
Se2—C56	1.923 (3)	C44—C45	1.381 (4)
N1—C11	1.375 (4)	C44—H44A	0.9500
N1—C1	1.476 (4)	C45—C46	1.393 (4)
N1—H1	0.883 (10)	C45—H45A	0.9500
N2—C51	1.389 (4)	C46—H46A	0.9500
N2—C2	1.469 (4)	C51—C52	1.394 (4)
N2—H2	0.893 (10)	C51—C56	1.415 (4)
C1—C31	1.530 (4)	C52—C53	1.385 (4)
C1—C21	1.547 (4)	C52—H52A	0.9500
C1—C41	1.557 (4)	C53—C54	1.381 (4)
C2—C61	1.554 (4)	C53—H53A	0.9500
C2—C81	1.543 (4)	C54—C55	1.383 (4)
C2—C71	1.547 (4)	C54—H54A	0.9500
C11—C12	1.395 (4)	C55—C56	1.387 (4)
C11—C16	1.417 (4)	C55—H55A	0.9500
C12—C13	1.370 (4)	C61—C66	1.375 (4)
C12—H12A	0.9500	C61—C62	1.396 (4)
C13—C14	1.384 (4)	C62—C63	1.399 (4)
C13—H13A	0.9500	C62—H62A	0.9500
C14—C15	1.379 (4)	C63—C64	1.374 (4)
C14—H14A	0.9500	C63—H63A	0.9500
C15—C16	1.399 (4)	C64—C65	1.369 (4)
C15—H15A	0.9500	C64—H64A	0.9500
C21—C26	1.387 (4)	C65—C66	1.408 (4)
C21—C22	1.389 (4)	C65—H65A	0.9500
C22—C23	1.379 (5)	C66—H66A	0.9500
C22—H22A	0.9500	C71—C72	1.379 (4)
C23—C24	1.391 (5)	C71—C76	1.394 (4)
C23—H23A	0.9500	C72—C73	1.381 (4)
C24—C25	1.366 (5)	C72—H72A	0.9500
C24—H24A	0.9500	C73—C74	1.386 (4)
C25—C26	1.385 (4)	C73—H73A	0.9500
C25—H25A	0.9500	C74—C75	1.375 (4)
C26—H26A	0.9500	C74—H74A	0.9500
C31—C32	1.387 (4)	C75—C76	1.380 (4)
C31—C36	1.389 (4)	C75—H75A	0.9500
C32—C33	1.389 (4)	C76—H76A	0.9500
C32—H32A	0.9500	C81—C86	1.381 (4)
C33—C34	1.380 (4)	C81—C82	1.395 (4)
C33—H33A	0.9500	C82—C83	1.380 (4)
C34—C35	1.387 (4)	C82—H82A	0.9500
C34—H34A	0.9500	C83—C84	1.366 (4)
C35—C36	1.374 (4)	C83—H83A	0.9500
C35—H35A	0.9500	C84—C85	1.368 (4)
C36—H36A	0.9500	C84—H84A	0.9500
C41—C46	1.387 (4)	C85—C86	1.392 (4)
C41—C42	1.382 (4)	C85—H85A	0.9500
C42—C43	1.387 (4)	C86—H86A	0.9500
C42—H42A	0.9500		
			
C16—Se1—Se2	103.03 (9)	C44—C43—H43A	120.1
C56—Se2—Se1	104.45 (9)	C45—C44—C43	120.1 (3)
C11—N1—C1	129.2 (3)	C45—C44—H44A	119.9
C11—N1—H1	118.4 (19)	C43—C44—H44A	119.9
C1—N1—H1	109.9 (19)	C44—C45—C46	119.6 (3)
C51—N2—C2	127.0 (3)	C44—C45—H45A	120.2
C51—N2—H2	115.5 (18)	C46—C45—H45A	120.2
C2—N2—H2	114.4 (18)	C41—C46—C45	120.9 (3)
N1—C1—C31	110.8 (3)	C41—C46—H46A	119.5
N1—C1—C21	109.6 (3)	C45—C46—H46A	119.5
C31—C1—C21	112.4 (3)	C52—C51—C56	117.8 (3)
N1—C1—C41	104.9 (2)	C52—C51—N2	122.8 (3)
C31—C1—C41	109.0 (3)	C56—C51—N2	119.3 (3)
C21—C1—C41	109.8 (3)	C51—C52—C53	120.6 (3)
N2—C2—C61	105.1 (2)	C51—C52—H52A	119.7
N2—C2—C81	109.8 (3)	C53—C52—H52A	119.7
C61—C2—C81	111.4 (2)	C52—C53—C54	121.6 (3)
N2—C2—C71	112.1 (3)	C52—C53—H53A	119.2
C61—C2—C71	104.0 (3)	C54—C53—H53A	119.2
C81—C2—C71	113.9 (3)	C55—C54—C53	118.4 (3)
C12—C11—N1	124.0 (3)	C55—C54—H54A	120.8
C12—C11—C16	117.4 (3)	C53—C54—H54A	120.8
N1—C11—C16	118.6 (3)	C54—C55—C56	121.3 (3)
C11—C12—C13	121.6 (3)	C54—C55—H55A	119.4
C11—C12—H12A	119.2	C56—C55—H55A	119.4
C13—C12—H12A	119.2	C51—C56—C55	120.2 (3)
C14—C13—C12	121.3 (3)	C51—C56—Se2	121.9 (2)
C14—C13—H13A	119.4	C55—C56—Se2	117.8 (2)
C12—C13—H13A	119.4	C66—C61—C62	118.3 (3)
C13—C14—C15	118.8 (3)	C66—C61—C2	123.0 (3)
C13—C14—H14A	120.6	C62—C61—C2	118.5 (3)
C15—C14—H14A	120.6	C61—C62—C63	121.0 (3)
C14—C15—C16	121.0 (3)	C61—C62—H62A	119.5
C14—C15—H15A	119.5	C63—C62—H62A	119.5
C16—C15—H15A	119.5	C64—C63—C62	119.9 (3)
C15—C16—C11	120.0 (3)	C64—C63—H63A	120.1
C15—C16—Se1	118.7 (2)	C62—C63—H63A	120.1
C11—C16—Se1	121.3 (2)	C65—C64—C63	119.8 (3)
C26—C21—C22	118.2 (3)	C65—C64—H64A	120.1
C26—C21—C1	122.8 (3)	C63—C64—H64A	120.1
C22—C21—C1	118.9 (3)	C64—C65—C66	120.6 (3)
C23—C22—C21	121.1 (4)	C64—C65—H65A	119.7
C23—C22—H22A	119.4	C66—C65—H65A	119.7
C21—C22—H22A	119.4	C61—C66—C65	120.4 (3)
C22—C23—C24	119.7 (4)	C61—C66—H66A	119.8
C22—C23—H23A	120.1	C65—C66—H66A	119.8
C24—C23—H23A	120.1	C72—C71—C76	118.1 (3)
C25—C24—C23	119.6 (4)	C72—C71—C2	119.2 (3)
C25—C24—H24A	120.2	C76—C71—C2	122.4 (3)
C23—C24—H24A	120.2	C71—C72—C73	121.4 (3)
C24—C25—C26	120.6 (4)	C71—C72—H72A	119.3
C24—C25—H25A	119.7	C73—C72—H72A	119.3
C26—C25—H25A	119.7	C74—C73—C72	120.0 (3)
C25—C26—C21	120.6 (3)	C74—C73—H73A	120.0
C25—C26—H26A	119.7	C72—C73—H73A	120.0
C21—C26—H26A	119.7	C73—C74—C75	119.2 (3)
C32—C31—C36	117.4 (3)	C73—C74—H74A	120.4
C32—C31—C1	120.4 (3)	C75—C74—H74A	120.4
C36—C31—C1	122.2 (3)	C74—C75—C76	120.7 (3)
C33—C32—C31	121.5 (3)	C74—C75—H75A	119.7
C33—C32—H32A	119.3	C76—C75—H75A	119.7
C31—C32—H32A	119.3	C71—C76—C75	120.6 (3)
C34—C33—C32	119.9 (3)	C71—C76—H76A	119.7
C34—C33—H33A	120.0	C75—C76—H76A	119.7
C32—C33—H33A	120.0	C86—C81—C82	117.6 (3)
C33—C34—C35	119.2 (3)	C86—C81—C2	124.3 (3)
C33—C34—H34A	120.4	C82—C81—C2	118.0 (3)
C35—C34—H34A	120.4	C83—C82—C81	120.6 (3)
C36—C35—C34	120.2 (3)	C83—C82—H82A	119.7
C36—C35—H35A	119.9	C81—C82—H82A	119.7
C34—C35—H35A	119.9	C82—C83—C84	120.9 (3)
C35—C36—C31	121.7 (3)	C82—C83—H83A	119.6
C35—C36—H36A	119.2	C84—C83—H83A	119.6
C31—C36—H36A	119.2	C85—C84—C83	119.5 (3)
C46—C41—C42	118.2 (3)	C85—C84—H84A	120.3
C46—C41—C1	119.1 (3)	C83—C84—H84A	120.3
C42—C41—C1	122.7 (3)	C84—C85—C86	120.2 (3)
C43—C42—C41	121.2 (3)	C84—C85—H85A	119.9
C43—C42—H42A	119.4	C86—C85—H85A	119.9
C41—C42—H42A	119.4	C81—C86—C85	121.1 (3)
C42—C43—C44	119.9 (3)	C81—C86—H86A	119.5
C42—C43—H43A	120.1	C85—C86—H86A	119.5
			
C16—Se1—Se2—C56	93.53 (14)	C42—C41—C46—C45	3.1 (5)
C11—N1—C1—C31	−85.7 (4)	C1—C41—C46—C45	−177.6 (3)
C11—N1—C1—C21	38.9 (4)	C44—C45—C46—C41	−1.9 (5)
C11—N1—C1—C41	156.7 (3)	C2—N2—C51—C52	10.7 (5)
C51—N2—C2—C61	177.4 (3)	C2—N2—C51—C56	−173.7 (3)
C51—N2—C2—C81	57.5 (4)	C56—C51—C52—C53	−2.0 (5)
C51—N2—C2—C71	−70.2 (4)	N2—C51—C52—C53	173.6 (3)
C1—N1—C11—C12	14.9 (5)	C51—C52—C53—C54	−0.9 (5)
C1—N1—C11—C16	−165.3 (3)	C52—C53—C54—C55	1.8 (5)
N1—C11—C12—C13	−179.7 (3)	C53—C54—C55—C56	0.3 (5)
C16—C11—C12—C13	0.5 (5)	C52—C51—C56—C55	4.1 (5)
C11—C12—C13—C14	0.9 (5)	N2—C51—C56—C55	−171.7 (3)
C12—C13—C14—C15	−1.0 (5)	C52—C51—C56—Se2	−179.8 (2)
C13—C14—C15—C16	−0.3 (5)	N2—C51—C56—Se2	4.4 (4)
C14—C15—C16—C11	1.8 (5)	C54—C55—C56—C51	−3.3 (5)
C14—C15—C16—Se1	179.7 (2)	C54—C55—C56—Se2	−179.6 (2)
C12—C11—C16—C15	−1.8 (4)	Se1—Se2—C56—C51	95.1 (3)
N1—C11—C16—C15	178.4 (3)	Se1—Se2—C56—C55	−88.7 (2)
C12—C11—C16—Se1	−179.7 (2)	N2—C2—C61—C66	−136.3 (3)
N1—C11—C16—Se1	0.5 (4)	C81—C2—C61—C66	−17.4 (4)
Se2—Se1—C16—C15	89.8 (2)	C71—C2—C61—C66	105.8 (3)
Se2—Se1—C16—C11	−92.3 (2)	N2—C2—C61—C62	48.3 (4)
N1—C1—C21—C26	−137.2 (3)	C81—C2—C61—C62	167.2 (3)
C31—C1—C21—C26	−13.4 (4)	C71—C2—C61—C62	−69.7 (3)
C41—C1—C21—C26	108.1 (3)	C66—C61—C62—C63	2.2 (5)
N1—C1—C21—C22	42.9 (4)	C2—C61—C62—C63	177.9 (3)
C31—C1—C21—C22	166.7 (3)	C61—C62—C63—C64	−0.3 (5)
C41—C1—C21—C22	−71.8 (4)	C62—C63—C64—C65	−1.3 (5)
C26—C21—C22—C23	1.3 (5)	C63—C64—C65—C66	1.1 (5)
C1—C21—C22—C23	−178.8 (3)	C62—C61—C66—C65	−2.4 (5)
C21—C22—C23—C24	−1.2 (5)	C2—C61—C66—C65	−177.9 (3)
C22—C23—C24—C25	0.0 (5)	C64—C65—C66—C61	0.8 (5)
C23—C24—C25—C26	1.1 (5)	N2—C2—C71—C72	−178.6 (3)
C24—C25—C26—C21	−1.0 (5)	C61—C2—C71—C72	−65.5 (3)
C22—C21—C26—C25	−0.2 (5)	C81—C2—C71—C72	56.0 (4)
C1—C21—C26—C25	179.9 (3)	N2—C2—C71—C76	−4.4 (4)
N1—C1—C31—C32	−162.2 (3)	C61—C2—C71—C76	108.6 (3)
C21—C1—C31—C32	74.7 (4)	C81—C2—C71—C76	−129.9 (3)
C41—C1—C31—C32	−47.2 (4)	C76—C71—C72—C73	1.8 (5)
N1—C1—C31—C36	18.5 (4)	C2—C71—C72—C73	176.3 (3)
C21—C1—C31—C36	−104.5 (3)	C71—C72—C73—C74	−0.4 (5)
C41—C1—C31—C36	133.5 (3)	C72—C73—C74—C75	−0.9 (5)
C36—C31—C32—C33	2.2 (5)	C73—C74—C75—C76	0.8 (5)
C1—C31—C32—C33	−177.1 (3)	C72—C71—C76—C75	−1.9 (5)
C31—C32—C33—C34	−0.2 (5)	C2—C71—C76—C75	−176.2 (3)
C32—C33—C34—C35	−2.3 (5)	C74—C75—C76—C71	0.6 (5)
C33—C34—C35—C36	2.9 (5)	N2—C2—C81—C86	−118.3 (3)
C34—C35—C36—C31	−0.9 (5)	C61—C2—C81—C86	125.7 (3)
C32—C31—C36—C35	−1.6 (5)	C71—C2—C81—C86	8.4 (4)
C1—C31—C36—C35	177.7 (3)	N2—C2—C81—C82	60.1 (3)
N1—C1—C41—C46	48.0 (4)	C61—C2—C81—C82	−55.9 (4)
C31—C1—C41—C46	−70.8 (3)	C71—C2—C81—C82	−173.2 (3)
C21—C1—C41—C46	165.7 (3)	C86—C81—C82—C83	−5.2 (5)
N1—C1—C41—C42	−132.7 (3)	C2—C81—C82—C83	176.2 (3)
C31—C1—C41—C42	108.6 (3)	C81—C82—C83—C84	3.8 (5)
C21—C1—C41—C42	−15.0 (4)	C82—C83—C84—C85	0.1 (5)
C46—C41—C42—C43	−1.6 (5)	C83—C84—C85—C86	−2.4 (5)
C1—C41—C42—C43	179.1 (3)	C82—C81—C86—C85	2.9 (5)
C41—C42—C43—C44	−1.2 (5)	C2—C81—C86—C85	−178.6 (3)
C42—C43—C44—C45	2.4 (5)	C84—C85—C86—C81	0.8 (5)
C43—C44—C45—C46	−0.9 (5)		

### Hydrogen-bond geometry (Å, º)

**Table:**

*D*—H···*A*	*D*—H	H···*A*	*D*···*A*	*D*—H···*A*
N1—H1···Se1	0.88 (1)	2.62 (3)	3.106 (3)	115 (19)
N2—H2···Se2	0.89 (2)	2.64 (2)	3.140 (2)	117 (2)
C35—H35*A*···Se1^i^	0.95	2.92	3.777 (3)	150

Symmetry code: (i) *x*−1, *y*, *z*.
